# Valproic Acid Induces Endothelial-to-Mesenchymal Transition-Like Phenotypic Switching

**DOI:** 10.3389/fphar.2018.00737

**Published:** 2018-07-11

**Authors:** Shamini Murugavel, Antoinette Bugyei-Twum, Pratiek N. Matkar, Husain Al-Mubarak, Hao H. Chen, Mohamed Adam, Shubha Jain, Tanya Narang, Rawand M. Abdin, Mohammad Qadura, Kim A. Connelly, Howard Leong-Poi, Krishna K. Singh

**Affiliations:** ^1^Faculty of Science, York University, Toronto, ON, Canada; ^2^Division of Cardiology, Li Ka Shing Knowledge Institute of St. Michael’s Hospital, Toronto, ON, Canada; ^3^Institute of Medical Science, University of Toronto, Toronto, ON, Canada; ^4^Vascular Surgery, Keenan Research Centre for Biomedical Science and Li Ka Shing Knowledge Institute of St. Michael’s Hospital, Toronto, ON, Canada; ^5^Department of Medicine, McMaster University, Hamilton, ON, Canada; ^6^Department of Pharmacology and Toxicology, University of Toronto, Toronto, ON, Canada; ^7^Department of Surgery, University of Toronto, Toronto, ON, Canada; ^8^Department of Medical Biophysics, Schulich School of Medicine and Dentistry, University of Western Ontario, London, ON, Canada

**Keywords:** valproic acid, endothelial cell, endothelial dysfunction, endothelial-to-mesenchymal transition, fibrosis

## Abstract

Valproic acid (VPA), a histone deacetylase (HDAC) inhibitor, is a widely used anticonvulsant drug that is currently undergoing clinical evaluation for anticancer therapy due to its anti-angiogenic potential. Endothelial cells (ECs) can transition into mesenchymal cells and this form of EC plasticity is called endothelial-to-mesenchymal transition (EndMT), which is widely implicated in several pathologies including cancer and organ fibrosis. However, the effect of VPA on EC plasticity and EndMT remains completely unknown. We report herein that VPA-treatment significantly inhibits tube formation, migration, nitric oxide production, proliferation and migration in ECs. A microscopic evaluation revealed, and qPCR, immunofluorescence and immunoblotting data confirmed EndMT-like phenotypic switching as well as an increased expression of pro-fibrotic genes in VPA-treated ECs. Furthermore, our data confirmed important and regulatory role played by TGFβ-signaling in VPA-induced EndMT. Our qPCR array data performed for 84 endothelial genes further supported our findings and demonstrated 28 significantly and differentially regulated genes mainly implicated in angiogenesis, endothelial function, EndMT and fibrosis. We, for the first time report that VPA-treatment associated EndMT contributes to the VPA-associated loss of endothelial function. Our data also suggest that VPA based therapeutics may exacerbate endothelial dysfunction and EndMT-related phenotype in patients undergoing anticonvulsant or anticancer therapy, warranting further investigation.

## Introduction

Valproic Acid or VPA, similar to other short chain fatty acids, is one of the well-known Histone Deacetylase (HDAC) inhibitors, which has been safely used for over 50 years as an anti-convulsant drug (Gottlicher et al., 2001; Phiel et al., 2001). VPA is shown to inhibit endothelial function and angiogenesis *in vitro* as well as *in vivo* (Michaelis et al., 2004; Kvestad et al., 2014). Angiogenesis is not only a physiological and critical process in vascular growth and wound healing (Flamme et al., 1997), it is also well characterized to play an integral and prominent role in cancer pathobiology (Bergers and Benjamin, 2003; Yadav et al., 2015). Therefore, anti-angiogenic therapies have been the spearhead of cancer therapeutics for decades (Ferrara and Kerbel, 2005; Jain, 2008; Heath and Bicknell, 2009), yet cancer largely remains incurable despite these advances in anti-angiogenesis research. VPA is also presented to be anti-angiogenic to alter angiogenicity in human cancers (Chelluri et al., 2016; Zhao et al., 2016), and is also currently undergoing clinical evaluation for anti-cancer therapy (Bezecny, 2014; Farooq et al., 2014; Yadav et al., 2015; Kwiecinska et al., 2016; Proske et al., 2016; Igarashi et al., 2017; Nilubol et al., 2017; Ramadoss et al., 2017).

The endothelium is made up of a single layer of endothelial cells (ECs) that configure along the lumen of all blood vessels (Dejana et al., 2017). This monolayer plays the role of a protective barrier in the space separating all tissues and the circulating blood. As a selective strainer, it is responsible to expedite the bidirectional travel of macromolecules and gasses to facilitate vascular homeostasis (Flammer and Luscher, 2010; Vita, 2011). One of the most important roles played by ECs is in the process of angiogenesis, which is a physiological process where from pre-existing vessels new blood vessels are formed during growth and development as well as during the process of wound healing (Chung and Ferrara, 2011). Angiogenesis is also a critical component in the transformation of tumors from a benign to a malignant state (Nishida et al., 2006). Furthermore, impaired angiogenesis contributes toward numerous ischemic, inflammatory, infectious and immune disorders (Carmeliet and Jain, 2011).

Apart from their role in angiogenesis, additionally ECs have the capability to transition into mesenchymal cells – this type of EC plasticity is known as endothelial-to-mesenchymal transition or EndMT (Goumans et al., 2008). This form is distinguished by the gain of mesenchymal or myofibroblastic phenotype with complementary loss of endothelial phenotype (Piera-Velazquez et al., 2011). EndMT is associated with gain of the mesenchymal markers such as neural (*N*)-Cadherin (*N*-Cadherin), fibroblast-specific protein 1 (FSP-1), αSMA, and types I/III collagen with corresponding loss of endothelial markers such as CD31, Tie-2 and vascular-endothelial (VE)-Cadherin (Piera-Velazquez et al., 2011). Aside from the acquisition of an activated pro-fibrogenic phenotype, ECs further lose their cell-cell junctions and achieve migratory and invasive capacity (Piera-Velazquez et al., 2011). EndMT is tightly regulated and known to play crucial roles in the process of development (Markwald et al., 1975; Wang et al., 2005), wound healing (Lee and Kay, 2006), and more recently has been involved in a broad range of pathological conditions such as cancer and organ fibrosis (Zeisberg et al., 2007a; Potenta et al., 2008). Zeisberg et al. (2007a) along with other reports (Potenta et al., 2008) provided convincing proof for EndMT-derived carcinoma-associated fibroblasts (CAFs) in the tumor microenvironment, where up to 40% of CAFs originated *via* EndMT. Zeisberg et al. (2007b) also confirmed the significant contribution of EndMT toward cardiac fibrosis. Later, Hashimoto et al. (2010) reported that, 16% of the lung fibroblasts from bleomycin (BLM)-treated mice (representative of pulmonary fibrosis) that were grown in culture had EC origin compared to 3% of those from saline-treated mice. Mechanistically, EndMT is thought to be instigated by inductive signals like TGFβs and β-catenin (Zeisberg et al., 2007b; Goumans et al., 2008; Medici et al., 2010, 2011). Wnt/β-catenin further interacts with TGFβ-signaling; VPA is correlated with the increased expression and activation of both TGFβ (Chelluri et al., 2016) and β-catenin (Lee et al., 2012) that induces EndMT (Yoshimatsu and Watabe, 2011; Wu et al., 2014).

Valproic acid is taken up by the endothelium immediately and crosses the blood-brain barrier within a minute of intravenous injection (Hammond et al., 1982). VPA has been shown to restrain angiogenesis *in vivo* and *in vitro* by inhibiting all basic aspects of angiogenesis (Michaelis et al., 2004; Gao et al., 2007; Isenberg et al., 2007; Shabbeer et al., 2007). VPA has also been shown to modulate TGFβ and β-catenin signaling (Lee et al., 2012; Chelluri et al., 2016); however, the direct effect of VPA on EC plasticity and EndMT remains undetermined. In the present study, we hypothesized that VPA-treatment leads to TGFβ and β-catenin signaling-mediated EndMT leading to loss of endothelial function *in vitro*. Accordingly, we demonstrate that VPA-treatment inhibits angiogenesis and proliferation. Additionally, for the first time, we demonstrate that VPA induces TGFβ-signaling-mediated EndMT-like phenotype switching *in vitro*, and upregulates vital genes involved in fibrosis. Our qPCR array data performed for 84 endothelial genes demonstrated 28 significantly and differentially regulated genes mainly implicated in angiogenesis, endothelial function, apoptosis, EndMT and fibrosis. These data suggest a completely novel and previously unknown role of VPA linking impaired angiogenesis, TGFβ-induced EndMT and up-regulation of pro-fibrotic genes. Given the important role played by both angiogenesis and EndMT in the induction of endothelial dysfunction, progression of cancer, as well as organ and cancer fibrosis, our data warrant future investigations.

## Materials and Methods

### Cell Culture and Valproic Acid Treatment

Human umbilical vein ECs (HUVECs, Lonza), human coronary artery ECs (HCAECs, Lonza) and human dermal microvascular ECs (HMVECs) were grown in EC growth medium-2 (EGM^TM^-2 Bulletkit^TM^; Lonza) containing growth factors or MCDB 131 (Gibco) supplemented with serum and antibiotics. After reaching 60–70% confluence, cells were starved over-night and then treated with 1, 2, 5, 10, and 20 mM of Valproic Acid (Santa Cruz Biotechnology). Control group were treated with the diluent. In order to determine the role played by TGFβ-signaling in our experimental setting, following starvation, HUVECs were pre-treated with 5 μM TGFβ-signaling inhibitor SIS3 (Calbiochem), which is a specific inhibitor of SMAD3 (Jinnin et al., 2006) for 2 h, prior to 5 mM of VPA treatment for an additional 24 h.

### *In Vitro* Angiogenesis Assay

The *In vitro* Angiogenesis Assay Kit (Chemicon) was employed to examine the effect of VPA on the angiogenic potential of ECs. ECs were seeded onto ECMatrix^TM^ Gel-coated 96-well plates at a cell density of 9 × 10^3^/well. The extent of angiogenesis was determined with Nikon phase contrast microscope, 2 h post-plating. Each experiment was performed thrice in triplicates.

### Nitric Oxide (NO) Quantification Assay

Cultured HUVECs were treated with 5 mM VPA or diluent and after 2 h of treatment, NO measurements were performed using Nitric Oxide Fluorometric Assay Kit (abcam) according to the manufacturer’s instructions.

### Proliferation and Migration Assay

Human umbilical vein endothelial cells were seeded at a density of 1 × 10^4^ cells/well in 96-well plates, treated with 5 mM VPA or diluent and cell proliferation was evaluated post-24 and 48 h treatment using WST-8 Cell Proliferation Assay Kit (Cayman Chemicals) according to the manufacturer’s instructions. Migratory capacity of HUVECs were evaluated using Cytoselect^TM^ 24-well Cell Migration and Invasion Assay kit according to the manufacturer’s protocol (Cell Biolabs, Inc).

### Quantitative Real Time PCR and PCR Array

Total RNA was isolated using Trizol^®^ (Invitrogen) method. Complementary DNA (cDNA) was produced using the Quantitect kit (Qiagen) and subjected to quantitative polymerase chain reaction (qPCR) with the ABI ViiA 7 Real-Time PCR System (Applied Biosystems). For the PCR reaction, SYBR^®^ Select Master Mix or TaqMan^®^ Gene Expression Assays (both Applied Biosystems) were mixed with forward and reverse primers for *CD31, VE-Cadherin, Tie2, αSMA, N-Cadherin, FSP1, Slug, TGFβ1, collagen I, CTGF, eNOS (endothelial nitric oxide synthase)* and *GAPDH* according to the manufacturer’s instructions as previously described (Singh et al., 2015). Primer sequences for eNOS, *Snail1, TFPI, cyclinD1, MMP-2, MMP-9 and p21* are described in the Supplementary Table 1. Quantitative real-time PCR analysis of 84 endothelial-related genes was accomplished using The Human Endothelial Cell Biology RT^2^ Profiler^TM^ PCR array (Qiagen). Data were analyzed as per the manufacturer’s integrated web-based software package. Validation qPCR performed for most up- and down-regulated genes *natriuretic peptides, brain type (BNP, catalog number # 4448892, GAPDH as control, catalog number # 4453320; both Thermofisher)* and *tissue factor pathway inhibitor (TFPI)*, respectively, confirmed their expression following VPA treatment. Validation qPCR was also performed for other relevant genes such as; *TGFβ1, Tie2, CD31, MMP-9 (matrix metalloproteinase 9) and MMP-*2 (Supplementary Table 1).

### Immunoblot and Immunofluorescence

Treated HUVECs were harvested 24 and 48 h post-treatment with either VPA or diluent and cell lysates were prepared in RIPA buffer (Sigma). Total protein was isolated and equal amounts of protein were loaded on sodium dodecyl sulfate (SDS) polyacrylamide gels. For immunoblotting analysis, the following primary antibodies were utilized at a 1:1000 dilution: CD31 (Cell Signaling #3528), VE-Cadherin (Santa Cruz Biotechnology #6458), Tie2 (Santa Cruz Biotechnology #324), *N*-Cadherin (abcam #ab76057), FSP1 (Abnova #H00006275-M01), αSMA (abcam #ab5694), α-actinin (Cell Signaling #3134), TGFβ1 (abcam #ab9758), SMAD2 (Cell Signaling #3122), pSMAD2 (Cell Signaling #3101), SMAD3 (abcam #ab28379 and Cell Signaling #9513), pSMAD3 (abcam #ab51451), SMAD5 (Cell Signaling #12534), pSMAD5 (abcam #ab92698), CTGF (abcam #ab6992), eNOS (Cell Signaling #9572), peNOS (Cell Signaling #9570), AKT (Cell Signaling #9272), pAKT (Cell Signaling #9271), β-catenin (Cell Signaling #8480), phospho-β-catenin (Cell Signaling #9561), and GAPDH (Millipore #MAB374). After final washes, the blot was developed with an enhanced chemiluminescence substrate (SuperSignal^TM^, Life Technologies) and a superior ChemiDoc^TM^ imaging system (Bio-Rad), and their intensities were quantified by densitometry using the ImageJ software. Immunofluorescence experiments were carried out in 4-chamber microscopy slides performed as previously described (Singh et al., 2015). Immunofluorescence signals from CD31, Tie2, αSMA and α-actinin staining were visualized with standard protocols 24 h post-treatment. Fluorescent microscopy images were captured using the Zeiss LSM700 confocal microscope and ZEN imaging software was utilized for image processing.

### Statistical Analysis

All data are expressed as mean ± SD unless otherwise specified. The Student’s *t*-test was applied when the means of two groups were being compared. Differences between multiple means were evaluated by ANOVA and, when overall differences were identified; individual means were compared *post hoc* with Bonferroni’s test. A *p*-value of <0.05 was considered to denote statistical significance.

## Results

### VPA Does Not Induce EC Apoptosis but Inhibits Angiogenesis

**FIGURE 1:**
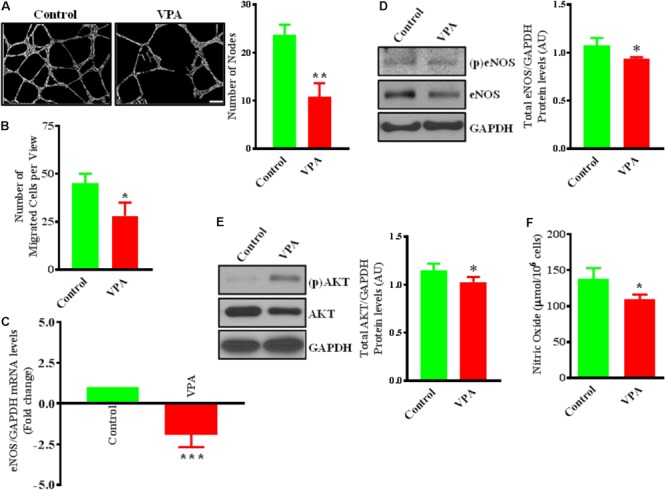
Valproic acid (VPA) impairs angiogenesis and negatively regulates eNOS/AKT expression and nitric oxide production in HUVECs. **(A)** Cultured HUVECs were transferred to a Matrigel-coated plate (BD Biosciences, San Jose, CA, United States) and treated with VPA or its diluent. Representative micrographs showing capillary-like tube formation in HUVECs 5 h after treatment with VPA. Tubular structures in 4 fields per group were semi-quantitatively analyzed. *N* = 4–5 in triplicate. ^∗∗^*p* < 0.01 vs. control group. **(B)** Bar graph demonstrating the migratory potential of cultured HUVECs after 12 h of VPA treatment. *N* = 4 in triplicate. ^∗^*p* < 0.05 vs. control group. **(C)** qPCR for eNOS and **(D)** immunoblot for eNOS, phospho (p)eNOS and quantification of eNOS on total RNA and protein extracted from HUVECs after 24 h of VPA treatment. *N* = 3 in triplicate. ^∗^*p* < 0.05, ^∗∗∗^*p* < 0.001 vs. control group. GAPDH was used as a loading control for immunoblot and internal control for qPCR. **(E)** Immunoblot for AKT, (p)AKT and quantification for AKT on total protein extracted from HUVECs after 24 h of VPA treatment. *N* = 3 in triplicate. ^∗^*p* < 0.05 vs. control group. **(F)** Bar graph demonstrating the nitric oxide production in cultured HUVECs after 6 h of VPA treatment. *N* = 10 in triplicate. ^∗^*p* < 0.05 vs. control group.

Valproic acid-treatment has been previously shown not to induce apoptosis in ECs (Michaelis et al., 2006). Accordingly, our immunoblotting data for cleaved-caspase-3 did not demonstrate any difference in the level of expression following 24 h of treatment with different therapeutically relevant concentrations (1, 2, 5, 10, and 20 mM) of VPA (Michaelis et al., 2004) (Supplementary Figure 1A). To evaluate whether endothelial function is also unaffected, we assessed the key indices of endothelial function *in vitro* and observed that the capacity of ECs to form capillary-like tubular structures was significantly reduced by VPA-treatment (**Figure 1A**). VPA also markedly attenuated the migratory capacity of ECs (**Figure 1B**). Our data were in accordance with previous reports where VPA was shown to reduce endothelial function *in vitro* (Michaelis et al., 2004). Collectively, these findings suggest an important role of VPA to limit the function and angiogenic potential of ECs *in vitro*.

### VPA Negatively Regulates eNOS/AKT Expression and Nitric Oxide Production

Endothelial cells constitutively express endothelial nitric oxide (NO)-synthase (eNOS), a key regulator of endothelial function (Deanfield et al., 2007). Our data also demonstrate that VPA significantly downregulated eNOS expression at transcript and protein level in ECs (**Figures 1C,D**). It is known that eNOS is a dynamic enzyme controlled by AKT-dependent phosphorylation at Ser^1177^ residue (Dimmeler et al., 1999; Sessa, 2004). Interestingly, VPA induced significant downregulation of AKT expression as well in ECs (**Figure 1E**). We did not observe any difference in the eNOS phosphorylation but AKT appear to be slightly phosphorylated by VPA treatment (**Figures 1D,E**). Reduced AKT and eNOS expression were further associated with reduced nitric oxide (NO) production by VPA-treated ECs (**Figure 1F**). These findings indicate that VPA inhibits both eNOS and AKT expression leading to decreased NO production, which are recognized regulators of endothelial function and angiogenesis.

### VPA Causes Marked Morphological and Ultrastructural Changes in ECs

**FIGURE 2:**
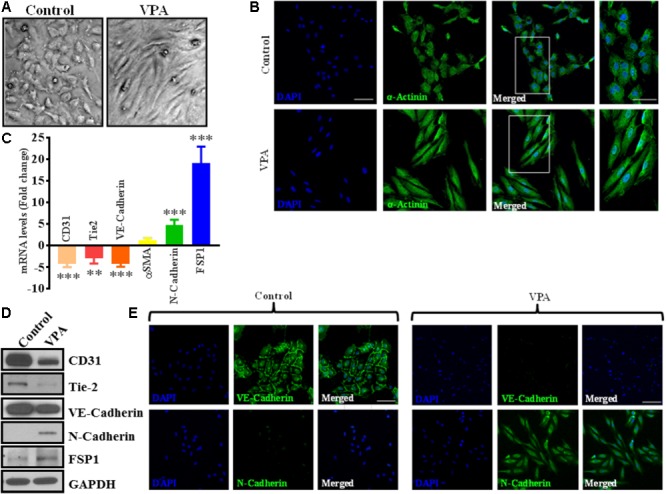
Valproic acid causes marked morphological and ultrastructural changes, and promotes EndMT-like phenotypic switching in HUVECs. **(A)** Diluent-treated control HUVECs, cultured on a two-dimensional plate, formed a confluent monolayer with the typical EC ‘cobblestone’ morphology (left panel). VPA treatment resulted in marked morphological changes whereby HUVECs took on an enlarged spindle-shaped appearance with smooth surfaces (right panel). Both micrographs were taken at the same magnification (10X). **(B)** Immunofluorescent micrographs demonstrating cytoskeletal protein re-organization in HUVECs following VPA treatment. α-Actinin positivity is indicated in green and nuclei were stained with DAPI (blue); scale bar = 10 μm. **(C)** HUVECs were treated with diluent control or VPA. Total RNA and protein were extracted at 24 and 48 h, respectively. Differential **(C)** transcript (qPCR) ^∗∗^*p* < 0.01, ^∗∗∗^*p* < 0.001 and **(D)** protein (immunoblotting) levels of key endothelial and mesenchymal markers as well as **(E)** VE-Cadherin and *N*-Cadherin immunofluorescent staining in control- and VPA-treated HUVECs indicate EndMT with VPA treatment. Nuclei were stained with DAPI (blue). Micrographs are representative images of HUVECs taken 48 h post-treatment; scale bar = 20 μm.

Notably, VPA-treated HUVECs, when observed under a light microscope, exhibited an obvious switch from the characteristic endothelial “cobblestone-like” manifestation to an enlarged spindle-shaped and smooth surfaced pattern that is consistent with “fibroblast-like” morphology (**Figure 2A**). These changes were complemented by an increase in α-actinin expression and mesenchymal cell-like cytoskeletal protein re-organization in VPA-treated HUVECs (**Figure 2B**).

### VPA Promotes Endothelial-to-Mesenchymal Transition-Like Phenotypic Switching

**FIGURE 3:**
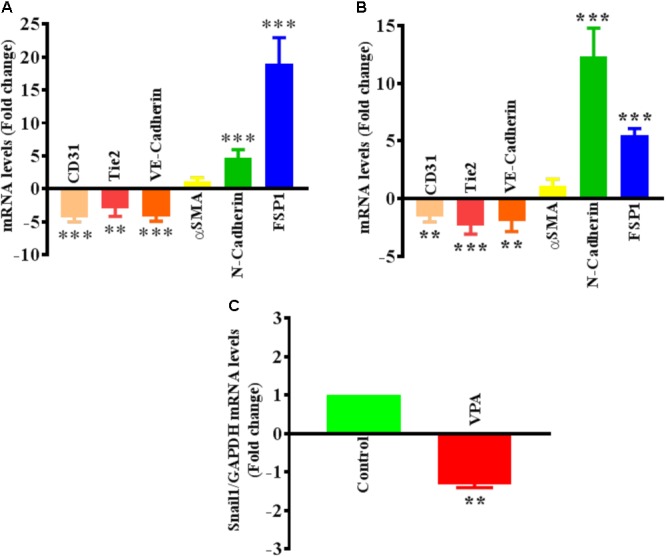
Valproic acid-associated EndMT is not specific to HUVECs. Cultured **(A)** HCAECs and **(B)** HMVECs were treated with 5 mM of VPA for 24 h and total RNA was extracted to perform the qPCR for EndMT markers. ^∗∗^*p* < 0.01, ^∗∗∗^*p* < 0.001 vs. corresponding control group. **(C)** Cultured HUVECs were treated with 5 mM of VPA for 24 h and total RNA was extracted to perform the qPCR for Snail1. ^∗∗^*p* < 0.01 vs. corresponding control group.

The differential transcript and protein levels of EC markers such as CD31, VE-Cadherin and Tie2, and mesenchymal markers such as αSMA, N-Cadherin and FSP1 in VPA-treated *vs.* control HUVECs is suggestive of the loss of endothelial, but gain of mesenchymal markers, consistent with EndMT features (**Figures 2C–E**). The vasculature is lined by a diverse population of ECs and there are variances between endothelium from different species, between large and small vessels, and between ECs derived from diverse microvascular endothelial beds. These variances are echoed in their ultrastructure, function, protein synthesis, and secretion (Jackson and Nguyen, 1997). As angiogenesis occurs in the microvasculature and not in large (macro) blood vessels such as veins, we also investigated whether the effect of VPA is unique to venous HUVECs. We conducted similar studies and measured the EndMT markers also in HCAECs and HMVECs, and confirmed *via* real-time PCR that VPA-treated HCAECs and HMVECs also displayed a similar EndMT-like phenotypic switching at molecular level (**Figures 3A,B**). The transcription factor Snail1 expression directly correlates with αSMA expression (Kokudo et al., 2008). Our data showed a significant down-regulation of Snail1 (**Figure 3C**), which appear to be associated with unaffected αSMA expression following VPA treatment to ECs (**Figures 2C, 3A,B**).

### VPA Activates TGFβ Signaling Pathway

**FIGURE 4:**
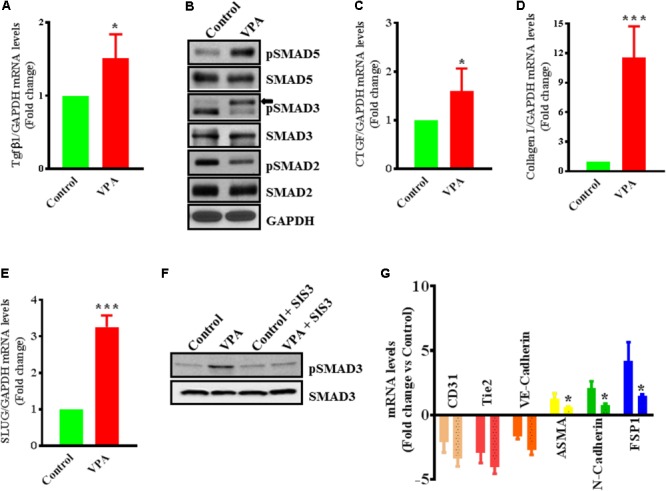
Valproic acid-associated EndMT occurs via a Tgfβ-dependent signaling pathways. HUVECs were treated with either diluent or VPA and total RNA and protein were extracted 24 h post-treatment to measure the TGFβ1 transcript **(A)** and phosphorylation of SMAD2/3/5 **(B)**. These changes were accompanied by up-regulation of **(C)** CTGF and **(D)** collagen I transcript levels ^∗^*p* < 0.05 and ^∗∗∗^*p* < 0.001. **(E)** VPA also induced SLUG expression in VPA-treated HUVECs. Cultured HUVECs were pre-treated with TGFβ-inhibitor SIS3 and then with VPA for 24 h. Later protein and RNA were extracted to confirm the TGFβ-inhibition *via* measuring pSMAD3 protein levels **(F)** and qPCR for EndMT marker levels **(G)**. In the bargraph the SIS3 + VPA group is shown by beaded bargraph. *N* = 3 in triplicates. ^∗^*p* < 0.05 vs. corresponding VPA group.

Endothelial-to-mesenchymal transition is thought to be instigated by inductive signals like TGFβs and β-catenin (Yoshimatsu and Watabe, 2011; Wu et al., 2014). Wnt/β-catenin interacts with TGFβ-signaling (Sun et al., 2015); VPA is associated with increased expression and translocation of β-catenin to the nucleus in human dermal papilla cell types (Lee et al., 2012) that induces EndMT in ECs, *via* suppression of endothelial markers (Cheon et al., 2004; Liebner et al., 2004). VPA is also shown to upregulate TGFβ1 expression in pericytes (Karen et al., 2011). Therefore, we postulated that VPA-treatment results in an increased Wnt/β-catenin and TGFβ-signaling leading to increased EndMT and increased expression of pro-fibrotic genes. In accordance, VPA-treated HUVECs had significantly higher TGFβ1 expression levels than controls (**Figure 4A**). TGFβ1 forms a complex with its receptors (Lijnen et al., 2000), goes on to phosphorylate SMAD proteins and relocates to the cell nucleus where it functions as a transcription factor for numerous TGFβ-dependent pro-fibrotic genes, such as CTGF and multiple collagens (Arciniegas et al., 1992; Lijnen et al., 2000; Goumans et al., 2008; Zeisberg and Neilson, 2009; Flammer and Luscher, 2010). Accordingly, VPA-treatment to HUVECs corresponded with increased SMAD3/5 phosphorylation (**Figure 4B**), signifying a molecular link between VPA and the TGFβ network. Increased TGFβ-signaling after VPA treatment to HUVECs were further associated with significantly increased expression of TGFβ-responsive pro-fibrotic genes; *CTGF* and *Collagen I* (**Figures 4C,D**). Furthermore, TGFβ-associated Slug production is known to play an essential role in TGFβ-induced EndMT (O’Riordan et al., 2007). Our data also demonstrated significantly increased Slug expression in the VPA-treated HUVECs in comparison to the control HUVECs (**Figure 4E**). These findings implicate VPA-induced TGFβ-signaling in the process of EndMT due to increased expression of TGFβ ligand (**Figure 4A**). Unexpectedly, the phosphorylation level of SMAD2 was reduced in VPA-treated ECs, however, SMAD2 and 3 are also known to be differentially activated by TGFβ (Liu et al., 2003), and both may also signal *via* independent pathways (Uemura et al., 2005). To further confirm the role of VPA-induced TGFβ-signaling toward EndMT, we inhibited TGFβ-signaling *via* inhibiting SMAD3 using pharmacological inhibitor SIS3. Our data on phosphorylation of SMAD3 following inhibition demonstrated reduced SMAD3 phosphorylation (**Figure 4F**) and diminished extent of EndMT in VPA-treated HUVECs (**Figure 4G**). These data further confirm the role of VPA-induced TGFβ-signaling in the process of EndMT.

We also examined the effect of VPA on β-catenin in ECs, as β-catenin expression and activation is a well-recognized trigger of EndMT (Wu et al., 2014). However, our data demonstrate that the VPA treatment to HUVECs led to slightly decreased expression and activation of β-catenin indicating reduced Wnt/β-catenin signaling (Supplementary Figures 1B–D). We further measured the expression level of β-catenin downstream target *cyclinD1* gene (Shtutman et al., 1999), which appears to be down-regulated after VPA treatment to HUVECs (mean ± SD fold-change -1.43 ± 0.20, *p* < 0.05 *vs.* control). These data show a differential and context-dependent effect of VPA on β-catenin signaling in different cell types and rules out its possible role in VPA-induced EndMT in HUVECs.

### VPA Up-Regulates Cyclin-Dependent Kinase Inhibitor p21 Expression and Inhibits Proliferation in ECs

**FIGURE 5:**
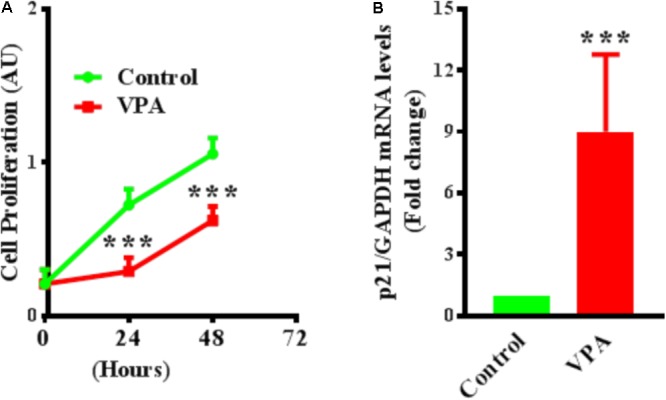
Valproic acid inhibits cell proliferation and up-regulates cyclin-dependent kinase inhibitor p21 expression in HUVECs. **(A)** Cultured HUVECs were treated with diluent or VPA and cell proliferation was evaluated at 0, 24, and 48 h post-VPA treatment. *N* = 6 in triplicates. ^∗∗∗^*p* < 0.001 vs. corresponding control group. **(B)** Bar graph representing the qPCR data for p21 performed on total RNA extracted from cultured HUVECs after 24 h of diluent or VPA treatment. *N* = 3 in triplicates. ^∗∗∗^*p* < 0.001 vs. corresponding control group.

Endothelial cell proliferation is an important aspect of endothelial function (Norton and Popel, 2016). VPA induced increased TGFβ1 expression and EndMT in ECs, whereas both TGFβ1 and EndMT, are associated with increased cell proliferation (Lebrin et al., 2004; Zeisberg et al., 2007b). Accordingly, we then tested whether VPA treatment is concomitant with enhanced cellular proliferation in HUVECs. Contrastingly, we witnessed a substantial decrease in cell proliferation following VPA treatment to the HUVECs (**Figure 5A**). Next, we evaluated the transcript and protein expression levels of cyclin-dependent kinase inhibitor p21, which was notably up-regulated following VPA treatment in ECs (**Figure 5B**). Since there was no significant difference in apoptosis following VPA-treatment to HUVECs (Supplementary Figure 1A), the VPA-induced p21 up-regulation appears to be the cause for reduced cell proliferation in HUVECs.

### qPCR Array Analysis of Endothelial-Related Genes in VPA vs. Control ECs Demonstrate Dysregulation of Several Endothelial Genes

A qPCR array for human endothelial-related genes was performed to further assess the effect of VPA on the expression level of various endothelial-related genes in ECs. As shown in **Tables 1, 2**, a total of 14 genes were significantly up- and 14 genes were significantly down-regulated, respectively, in the VPA-treated ECs. The most up-regulated genes include *BNP* (*Natriuretic peptide B*; 145-fold) and *MMP-9* (*Matrix metallopeptidase 9*, 121-fold) (**Table 1**). The most down-regulated genes were *TFPI* (*Tissue factor pathway inhibitor*, 5.8-fold) and *SPHK* (Sphingosine kinase 1, 5-fold) (**Table 2**). Validation qPCR was performed for the most up- and down-regulated genes, *BNP* (mean ± SD fold-change 45 ± 8, *p* < 0.0001 *vs.* control) and *TFPI* (mean ± SD fold-change 15 ± 3, *p* < 0.001 *vs.* control), respectively, which demonstrated the similar expression pattern as observed in qPCR array following VPA treatment. Validation qPCR was also performed for other relevant significantly up- [*MMP-9* (mean ± SD fold-change 18 ± 5, *p* < 0.001 *vs.* control), and *TGFβ1* (mean ± SD fold-change 2.3 ± 0.3, *p* < 0.01 *vs.* control)] and down-regulated genes [*MMP-2* (mean ± SD fold-change -1.6 ± 0.2, *p* < 0.01 *vs.* control), *Tie-2* (mean ± SD fold-change -3.5 ± 0.23, *p* < 0.001 *vs.* control) and *CD31* (mean ± SD fold-change -3.3 ± 0.31, *p* < 0.01 *vs.* control)], which also demonstrated the similar expression pattern as observed in the qPCR array (**Tables 1, 2**).

**Table 1:** Endothelial-related genes PCR array.

No.	Up-regulated genes	Gene name	Fold-regulation
1	BNP	Natriuretic peptide B	145.0762*
2	MMP-9	Matrix metallopeptidase 9	121.0115***
3	PF-4	Platelet factor 4	25.223***
4	T-PA	Plasminogen activator, tissue	18.4132*
5	5-LOX	Arachidonate 5-lipoxygenase	16.3324**
6	TP	Thymidine phosphorylase	14.261**
7	PDGFR-2	Platelet-derived growth factor receptor	11.6722**
8	CXCL3	Chemokine (C-X3-C motif) ligand 1	8.4894*
9	COX-2	Prostaglandin-endoperoxide synthase 2	3.4726*
10	LSEL	Selectin L	3.1616***
11	TIMP	TIMP metallopeptidase inhibitor 1	1.7666*
12	APO-E	Apolipoprotein E	1.5273*
13	EPCR	Protein C receptor, endothelial	1.2681**
14	TGFβ	Transforming growth factor, beta 1	1.2681*

*Genes up-regulated in VPA-treated vs. control ECs. ^∗^*p* < 0.05, ^∗∗^*p* < 0.01, and ^∗∗∗^*p* < 0.001 vs. control group.*

**Table 2:** Endothelial-related genes PCR array.

No.	Down-regulated genes	Gene name	Fold-regulation
1	TFPI	Tissue factor pathway inhibitor	–5.8685^∗∗^
2	SPHK	Sphingosine kinase 1	–5.0771^∗∗^
3	FAS1	TNF receptor superfamily, member 6	–4.4878^∗^
4	PLGF	Placental growth factor	–3.9132^∗∗^
5	TIE-2	TEK tyrosine kinase, endothelial	–3.4943^∗^
6	CGL3	Caveolin 1, caveolae protein, 22kDa	–3.2595^∗^
7	CASP8AP1	CASP8 and FADD-like apoptosis regulator	–2.8613^∗∗^
8	IL-6	Interleukin 6 (interferon, beta 2)	–2.8284^∗∗^
9	CD31	Platelet/endothelial cell adhesion molecule	–2.484^∗^
10	CPP32	Caspase 3, cysteine peptidase	–2.0378^∗^
11	ITGA5	Integrin, alpha 5	–1.5562^∗^
12	MMP-2	Matrix metallopeptidase 2	–1.4746^∗^
13	KNO	Collagen, type XVIII, alpha 1	–1.4119^∗^
14	BCL2L1	BCL2-like 1	–1.3007^∗^

*Genes down-regulated in VPA-treated vs. control ECs. ^∗^p < 0.05, ^∗∗^p < 0.01 and ^∗∗∗^p < 0.001 vs. control group.*

## Discussion

The main observation made in this study is that VPA leads to TGFβ-signaling mediated EndMT-like phenotypic switching causing up-regulation of pro-fibrotic genes and dysregulation of several regulatory endothelial genes culminating in loss of endothelial function. Given VPA’s prominent clinical application, for the first time our data indicate that its use might exacerbate the known EndMT and loss of endothelial function associated human pathologies, and warrant investigations.

Although the anti-angiogenic effect of VPA has been largely studied, we further evaluated the anti-angiogenic effects of VPA on ECs. First, we asked whether VPA induces apoptosis in HUVECs and evaluated apoptosis using clinically relevant (1, 2, and 5 mM) (Michaelis et al., 2004) and high (10 and 20 mM) doses of VPA. Our immunoblotting data for cleaved-caspase-3 did not show induction of apoptosis in HUVECs following VPA-treatment (Supplementary Figure 1A). To evaluate endothelial function, we assessed the key indices of endothelial function such as tube formation and migration *in vitro* and observed that the capacity of ECs to form capillary-like tubular structures as well as the migratory capacity was significantly reduced by VPA-treatment (**Figures 1A,B**). These data were also in accordance with previous reports; where VPA was shown to reduce endothelial function (Michaelis et al., 2004). Collectively, our findings suggest an important role of VPA to limit the function and angiogenic capacity of the ECs *in vitro*. ECs constitutively express eNOS, a key regulator of endothelial function that is controlled by AKT-dependent phosphorylation (Dimmeler et al., 1999; Sessa, 2004). VPA significantly downregulated total eNOS and total AKT expression (**Figures 1C–E**). Phosphorylation of eNOS appeared to be unaffected but there was slight increase in the phosphorylation of AKT in 5 mM of VPA-treated ECs in comparison to controls (**Figures 1D,E**). However, previously, Michaelis et al. (2004) did not detect any effect of low dose of VPA (1 mM) on AKT or eNOS expression and phosphorylation in HUVECs. Reduced eNOS expression was further associated with reduced nitric oxide (NO) production by VPA-treated ECs (**Figure 1F**). This is in accordance with previous data; HDAC inhibitors such as Trichostatin A, NaBu, and MS-275 were associated with reduced eNOS expression and NO generation in ECs (Rossig et al., 2002). VPA has also been shown to reduce the expression level of AKT1 by PCR array in cancer cells (Chelluri et al., 2016) but its effect in ECs has not been reported. HDAC inhibition leads to the induction of eNOS mRNA destabilizing protein, causing a decrease in eNOS mRNA and protein expression (Rossig et al., 2002). Michaelis et al. reported reduction in eNOS using 1 mM of VPA after 3 days. VPA can inhibit the activation of AKT and proliferation of HeLa cells and SiHa cells, in a concentration dependent (0, 0.5, 1, 2, 4, 8, and 16 mM) manner (Zhao et al., 2016). These findings indicate that VPA inhibits eNOS and AKT expression leading to decreased NO production, which are the recognized regulators of endothelial function and angiogenesis. Finally, in the milieu of cardiovascular risk factors that disturb vascular homeostasis, the endothelium becomes dysfunctional resulting in perturbed angiogenesis, which is one of the most important phenotypes either as a cause or effect for cardiovascular diseases (CVDs) – the number one cause of death globally (Source: fact sheet, WHO). Given the higher prevalence of CVDs, these data warrant the need for studies to conclude the effect of VPA-treatment on CVDs in addition to its anti-epileptic or anti-cancer use.

Under stressful and pathophysiological scenarios, ECs have displayed a noteworthy amount of plasticity. However, there has been no report linking VPA to the maintenance of cellular architecture, phenotypic transformation, and/or mesenchymal transition. Although EndMT was initially described during embryonic cardiogenesis, it also occurs in the post-natal state, particularly during the development of organ fibrosis, pulmonary vein stenosis, anomalous vascular remodeling, cerebral cavernous malformations, and cancer progression (Medici et al., 2010; Nataraj et al., 2010; Chen et al., 2012; Maddaluno et al., 2013; Kato et al., 2014). A trademark of these phenomena is generally a loss of endothelial and gain of mesenchymal and stem-cell like markers. The TGFβ pathway is the primary mediator of EndMT, which is facilitated through the phosphorylation of SMAD proteins and subsequent transcription of key target genes (Zeisberg et al., 2007b). Induction of β-catenin is also known to induce EndMT, and Wnt/β-catenin interaction further aggravates this EndMT phenotype (Cheon et al., 2004). Identifying the biological cues that control EndMT can therefore provide significant insights into the VPA-treatment-associated pathophysiology, in addition to organ fibrosis and cancer progression.

There are enough data indicating that VPA inhibits angiogenesis, activates modulators of EndMT such as TGFβ and β-catenin, and inhibits mesenchymal-like phenotype in pericytes but its direct and precise role in EndMT in ECs is not evaluated. Here, for the first time, we demonstrate the effect of VPA on EndMT-like phenotypic switching in ECs. Interestingly, in HUVECs VPA promoted EndMT, in conjunction with evident morphological and ultra-structural variations from “cobblestone-like EC morphology” to an enlarged spindle shaped, smooth surfaced “fibroblast like morphology” (**Figure 2A**). These morphological alterations were in parallel to the cytoskeletal protein re-organization as confirmed by α-actinin staining (**Figure 2B**). The “fibroblast like morphology” was further linked to the reduced expression of the endothelial markers CD31, VE-Cadherin, and Tie2, and augmented expression of the mesenchymal markers N-Cadherin, FSP1 and Slug (**Figures 2C–E**). Although the αSMA expression is also an important hallmark of EndMT (Ghosh et al., 2012), surprisingly, we did not observe a significant change in αSMA expression level after VPA treatment to HUVECs (**Figure 2C**). Furthermore, the transcription factor Snail1 expression directly correlates with αSMA expression (Kokudo et al., 2008) and VPA-treatment caused a significant down-regulation of Snail in ECs (**Figure 3C**). Given the direct and negative effect of VPA on Snail expression, it is plausible that VPA-induced inhibition of Snail is the cause behind un-affected αSMA expression following VPA-treatment in ECs. Additionally, given the known TGFβ-Akt-Snail-axis in EndMT (Widyantoro et al., 2010), VPA-induced inhibition of AKT and Snail might be another explanation for observed un-affected αSMA expression in ECs after VPA-treatment.

Moreover, angiogenesis happens in the microvasculature and not in large blood vessels such as veins. To confirm that the effect of VPA is not unique to venous HUVECs, we performed parallel studies and measured the EndMT markers also in HCAECs and HMVECs. We confirmed *via* real-time PCR that VPA-treated HCAECs and HMVECs also displayed a similar EndMT-like phenotypic switching at molecular level (**Figures 3A,B**). Patterns of αSMA expression in HCAECs and HMVECs also followed the similar trend as in HUVECs after VPA-treatment (**Figures 2C, 3A,B**).

Mechanistically, the levels of acetylated histone H4 in the absence and presence of VPA is very well evaluated, where 1mM of VPA significantly enhances acetylated histone H4 (Michaelis et al., 2004). Accordingly, we also observed increased activation of acetylated histone H4 in HUVECs following VPA treatment (Supplementary Figure 1B). However, EndMT is thought to be initiated by inductive signals like TGFβs and Wnt/β-catenin, and reports suggest an interaction between TGFβ and Wnt-signaling pathways in the induction of EndMT (Liebner et al., 2004). Wnt/β-catenin interacts with TGFβ-signaling; VPA is associated with increased expression of TGFβs and its effectors (Karen et al., 2011), and expression and activation of β-catenin (Lee et al., 2012) that induces EndMT (Cheon et al., 2004). Accordingly, VPA-treated HUVECs had significantly higher TGFβ1 transcript levels than controls (**Figure 4A**). TGFβ1 forms a complex with its receptors (Lijnen et al., 2000), phosphorylates SMAD proteins, and relocates to the nucleus to function as a transcription factor for various TGFβ-dependent pro-fibrotic genes, such as CTGF and collagens (Arciniegas et al., 1992; Lijnen et al., 2000; Goumans et al., 2008; Zeisberg and Neilson, 2009; Flammer and Luscher, 2010). Furthermore, TGFβ-related Slug plays an essential role in TGFβ-induced EndMT (O’Riordan et al., 2007). VPA-treatment to HUVECs corresponded with increased SMAD3/5 phosphorylation (**Figure 4B**), advocating a molecular link between VPA and the TGFβ web. TGFβ, TGFβ-responsive pro-fibrotic genes; such as CTGF and Collagen I, were also considerably up-regulated in the VPA-treated HUVECs when compared to the control HUVECs (**Figures 4C,D**). Our results reveal that VPA-associated increased TGFβ activity in HUVECs is possibly a result of the augmented transcription of TGFβ ligand and Slug expression leading to EndMT (**Figure 4E**). However, to further confirm the contribution of VPA-induced TGFβ-signaling toward EndMT, we inhibited TGFβ-signaling *via* pharmacologically inhibiting SMAD3, which significantly diminished the extent of EndMT in VPA-treated HUVECs (**Figures 4F,G**) confirming the contribution of TGFβ-signaling in VPA-associated EndMT. However, it is well described that SMAD3 transduce signals for TGFβ, while SMAD5 is specific for BMP signaling (Zi et al., 2012). Given the up-regulation of SMAD5 following VPA-treatment, it appears that BMP signaling might also contribute toward VPA-associated endothelial phenotype. VPA-induced Wnt/β-catenin signaling causes increased expression, accumulation and activation of β-catenin in Neuro2A cells (Phiel et al., 2001). Furthermore, β-catenin translocation to the nucleus is a known trigger of EndMT (Wu et al., 2014). We, therefore examined the effect of VPA on β-catenin in ECs. Unexpectedly, our data demonstrated that the VPA treatment to the HUVECs led to a slightly decreased expression and activation of β-catenin (Supplementary Figures 1B–D). We next measured the expression level of β-catenin downstream target *cyclinD1* gene (Shtutman et al., 1999), which was significantly down-regulated after VPA treatment in HUVECs but it can also be a β-catenin-independent effect (Sansom et al., 2005). Taken together, these data demonstrate the cell-type and context-dependent activation of Wnt/β-catenin signaling by VPA in different cell types and rule out the possible role of Wnt/β-catenin signaling in VPA-induced EndMT.

Furthermore, EC proliferation is an important aspect of endothelial function. VPA induced increased TGFβ1 expression and EndMT in ECs, where both TGFβ1 and EndMT, are associated with increased cell proliferation (Lebrin et al., 2004; Zeisberg et al., 2007b). Accordingly, we next tested whether VPA treatment is related to the increased cell proliferation in HUVECs. Contrastingly, we observed a significant decrease in the cell proliferation following VPA treatment to the HUVECs (**Figure 5A**). Our results seem to be in line with other researchers, where VPA-treatment is shown to reduce proliferation in tumors (Michaelis et al., 2004; Chelluri et al., 2016). Mechanistically, TGFβ target cytostatic genes such as expression level of cyclin-dependent kinase inhibitor p21 (Cordenonsi et al., 2003; Cordenonsi et al., 2007) and TGFβ-mediated induction of p21 has been previously reported specifically in endothelial cells (Bai et al., 2017). Therefore, next we evaluated the expression level of *p21*, which was significantly up-regulated following VPA treatment to ECs (**Figure 5B**), which appears to be TGFβ-mediated. Since, MTT assay cannot distinguish between cytotoxic and cytostatic effect, results were also confirmed by immunoblotting for cleaved-caspase-3 (Supplementary Figure 1A). Our data indicate that VPA was not cytotoxic and the observed effect could be credited to the inhibition of p21-mediated cell proliferation. The observed reduced migration following VPA treatment (**Figure 1B**) can be attributed to the reduced EC proliferation.

To evaluate the overall effect of VPA on endothelial-related gene expression in HUVECs, a qPCR array for 84 human endothelial-related genes was performed. As shown in **Tables 1, 2**, a total of 14 genes were significantly up-regulated and 14 genes were significantly down-regulated, respectively, in the VPA treated ECs. It is interesting to note that the most-upregulated gene *BNP*, is a hormone secreted by cardiomyocytes in the heart and is typically increased in patients with left ventricular dysfunction and thereby being used for screening as well as for the prognosis of the heart failure (Atisha et al., 2004; Bhalla et al., 2004). Although BNP is mainly secreted by cardiomyocytes, its expression has also been reported in other cell-types including ECs of macro- and micro-circulation in response to stressors such as hypoxia, and known to preferably exert more paracrine than endocrine effects (Bordenave et al., 2002; Kuhn, 2012). There is no literature available on the direct role of BNP on angiogenesis, endothelial function and/or EndMT but it has been shown to relax vascular smooth muscle in arterioles and venules (Cui et al., 2012). However, in the heart following injury BNP facilitates neutrophil infiltration and increases the MMP-9 activity, which is the second most up-regulated gene in the qPCR array after VPA treatment to HUVECs. Furthermore, MMP-9 appears to be critical for TGFβ-mediated EndMT differentiation of endothelial to CAF-like cells (Ciszewski et al., 2017). This suggests that BNP and MMP-9 both play role in the processes of extracellular matrix remodeling and wound-healing (Nakatani et al., 2018), which is related to EndMT and fibrosis (Chen and Frangogiannis, 2013). Among the other up-regulated genes, most of up-regulated genes such as, PF-4 (Schwarz et al., 2003), T-PA (Ghosh and Vaughan, 2012), 5-LOX (Oak et al., 2014), COX-2 (Oak et al., 2014; Zhang et al., 2014), PDGFR-2 (Saito et al., 2017), CXCL-3 (Mukaida et al., 2014), LSEL (Hamaguchi et al., 2002), and TGFβ1 (Pohlers et al., 2009) are known to play a direct role in the progression of fibrosis. Furthermore, VPA has been shown to demonstrate anti-cancer activity in breast cancer *via* induction of TP or thymidine phosphorylase expression (Terranova-Barberio et al., 2016). The up-regulation of TP contributes to angiogenesis, which may also play a critical role in the progression of fibrosis (Wang et al., 2006). Interestingly, in our qPCR array, we also observed significantly increased expression of TP in ECs following VPA treatment. TIMP-1 was the only up-regulated gene that directly correlates with endothelial dysfunction (de la Sierra and Larrousse, 2010).

*Tissue Factor Pathway Inhibitor* or *TFPI*, which is a natural coagulation inhibitor (Nordfang et al., 1991), was the most down-regulated gene identified by our qPCR array. It is interesting to note that the VPA has already been shown to have a protective effect in severe hemorrhage and ischemia-reperfusion injury (Causey et al., 2012, 2013). Reduced TFPI expression following VPA treatment to ECs might be the mechanism behind the beneficial phenotype observed in severe hemorrhage, however, it needs to be confirmed (Causey et al., 2013). Most of the other down-regulated genes such as, SPHK (Pham et al., 2010), FAS-1 (Gerber et al., 1998), PLGF (Bottomley et al., 2000), Tie-2 (Dejana et al., 2017), CGL3 (Fernandez-Hernando et al., 2010), CD31 (Dejana et al., 2017), MMP-2 (Taraboletti et al., 2002), and KNO (Passos-Bueno et al., 2006) are known to be pro-angiogenic in nature and required for efficient endothelial function. Interestingly *CASP8AP1* (Viemann et al., 2007) and BCL2L1 (Karsan et al., 1997), both are known to play a role in endothelial apoptosis were down-regulated following VPA treatment to ECs.

It is also interesting to note that two matrix metalloproteinases MMP-2 and MMP-9 demonstrated an inverse correlation following VPA treatment, and there is a report correlating different levels of MMP-2 and MMP-9 with different outcomes in humans (Tabouret et al., 2016), however, their interplay and the effect on ECs following VPA treatment needs to be further investigated. These significantly differentially expressed genes in HUVECs in response to VPA provide the basis for possible mechanisms leading to observed increased TGFβ-signaling, EndMT, increased pro-fibrotic genes, reduced angiogenesis, un-affected apoptosis and endothelial dysfunction.

Taken together, our data clearly demonstrate that VPA-treatment to ECs induces TGFβ-signaling mediated EndMT, up-regulates pro-fibrotic genes and down-regulates pro-angiogenicity leading to loss of endothelial function. Accordingly, our data warrants further investigation of VPA as an anti-psychotic or anti-cancer therapy to avoid the exacerbation of fibrosis or endothelial dysfunction-related outcomes in patients. There appear to be an immense need to gain a better understanding of the molecular underpinnings to find a fine balance between anti-angiogenic and pro-fibrotic potential of VPA as they may offer clues for better potential translational evaluation leading to differential diagnostic and treatment and/or personalized therapy. This newly discovered biological function might help to explain the different and context-dependent effects observed in the patient population. Future *in vivo* studies using diverse animal models of human pathologies will be necessary to further clarify the effect of VPA on ECs and the potential for its optimal therapeutic use.

## Author Contributions

KKS conceived and designed the study. KKS, AB-T, PM, HC, HA-M, SM, RA, SJ, TN, and MA carried out the experiments and analyzed the data. HL-P, KC, and MQ helped write and improve the discussion. KKS and SM wrote and assembled the manuscript. KKS, AB-T, PM, and HC helped with final figures and in finalizing of the manuscript.

## Conflict of Interest Statement

The authors declare that the research was conducted in the absence of any commercial or financial relationships that could be construed as a potential conflict of interest.

## Abbreviations

αSMA: alpha smooth muscle actin
CTGF: connective tissue growth factor
EC(s): endothelial cell(s)
EndMT: endothelial-to-mesenchymal transition
FSP1: fibroblast-specific protein-1
H&E: hematoxylin and eosin
HUVECs: human umbilical vein endothelial cells
N-cadherin: neural cadherin
pSMAD: phosphorylated SMAD
qPCR: quantitative RNA polymerase chain reaction
TGFBR: transforming growth factor receptor type
