# Supplementary material for: Valproic Acid Induces Endothelial-to-Mesenchymal Transition-Like Phenotypic Switching
Source: Front Pharmacol. 2018 Jul 11;9:737. doi: 10.3389/fphar.2018.00737 (PMC6050396; doi:10.3389/fphar.2018.00737)
Supplement: Supplementary file 1 [file Table_1.PDF]

Supplementary Table 1

Genes	Nucleotide Sequences
p21- Forward	5'-agtcagttccttgtggagcc-3'
p21- Reverse	5'-catgggttctgacggacat-3'
eNOS- Forward	5'-tgagtatgacgtggtgtccc-3'
eNOS- Reverse	5'-gacatctccatcagggcag-3'
cyclinD1- Forward	5'-tgtcctactaccgcctcaca-3'
cyclinD1- Reverse	5'-cagggcttcgatctgctc-3'
Snail1- Forward	5'-gcctagcgagtggttcttct-3'
Snail1- Reverse	5'-tagggctgctggaaggtaaa-3'
TFPI- Forward	5'-tgcatccttcccatcagtatta-3'
TFPI- Reverse	5'-gtgagttctgggagcattttg-3'
MMP-9- Forward	5'-gaaccaatctcaccgacagg-3'
MMP-9- Reverse	5'-gccacccgagtgtaaccata-3'
MMP-2- Forward	5'-cccaaaacggacaaagagtt-3'
MMP-2- Reverse	5'-caaacaggttgcagctctcc-3'

List of primers used to perform qPCR.
