# Supplementary material for: Valproic Acid Induces Endothelial-to-Mesenchymal Transition-Like Phenotypic Switching
Source: Front Pharmacol. 2018 Jul 11;9:737. doi: 10.3389/fphar.2018.00737 (PMC6050396; doi:10.3389/fphar.2018.00737)
Supplement: Supplementary file 2 [file Data_Sheet_1.PDF]

SUPPLEMENTARY FIGURE 1

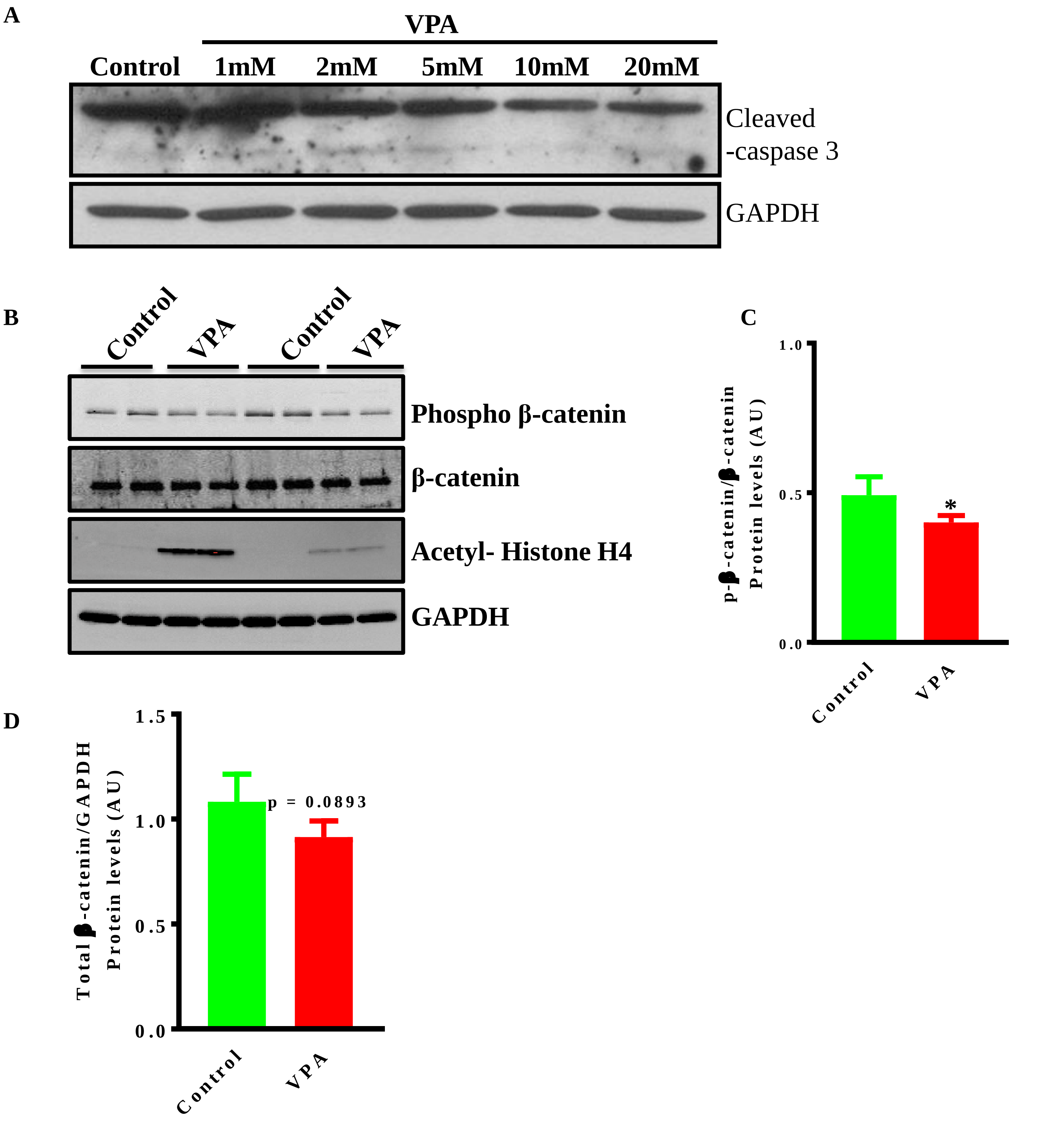

Supplementary Figure 1. (A) Cultured HUVECs were treated with different concentration (1, 2, 5, 10 and 20mM) of VPA and total protein was isolated 24 hours post-treatment. Immunoblot was performed for **(A)** cleaved-caspase 3 and **(B)** β-catenin, phospho β-catenin and acetyl- histone H4 was performed. GAPDH was used as a loading control. Quantification of the ratio of phospho β-catenin/ total β-catenin **(C)** and total β-catenin/GAPDH **(D)**. **p<0.05 versus corresponding control group.
